# Isolated First Web Space Palmar Fibroproliferation in an Avid Pistol Shooter: A Case Report

**DOI:** 10.7759/cureus.100201

**Published:** 2025-12-27

**Authors:** Karter Morris, Ty Kaatz, Cameron T Cox, Evan J Hernandez, Brendan J MacKay

**Affiliations:** 1 School of Medicine, Texas Tech University Health Sciences Center, Lubbock, USA; 2 Orthopedic Surgery and Rehabilitation, Texas Tech University Health Sciences Center, Lubbock, USA

**Keywords:** dupuytren’s contracture, fibroproliferative disorders, hand deformity, occupational hand injury, palmar aponeurosis

## Abstract

There are various types of contractures of the hand, including diseases of fibroproliferation. However, instances of isolated, atraumatic contractures of the first web space are not well described in the literature. A 65-year-old avid pistol shooter presented to the clinic with a unilateral deformity of his dominant hand. The abnormality developed since he started regularly shooting pistols around one year prior, and it has not caused any pain or functional deficits. There was no relevant medical or family history. On physical examination, the patient exhibited a visible, palpable cord in line with the lateral margin of the palmar aponeurosis. The cord was non-mobile, and differential diagnoses included Dupuytren’s contracture and stenosing tenosynovitis. Ultrasound revealed asymmetrical thickening of the palmar aponeurosis in the first web space, and a descriptive working diagnosis of “pistol shooter’s palm” was considered. The patient was monitored clinically, and surgical intervention was not recommended. The etiology of hand contractures often goes unaccounted for; however, this case describes a potential contributing factor associated with an abnormal presentation of fibroproliferation in the hand.

## Introduction

Hand contractures are a well-documented disease process due to fibrous proliferation that results in the deposition of collagen [1, 2]. This abnormal fibroproliferation can lead to impaired hand mobility, strength, and overall function. Dupuytren’s contracture is one of the more commonly known hand contractures. The exact etiology is unknown but is believed to be of primarily genetic etiology that precedes disease development [3,4]. Dupuytren’s contracture typically involves the fourth and fifth webspace of the palmar fascia but may affect any digit [3,4]. Approximately 80% of the disease variance can be attributed to genetic factors [5]. The remaining 20% of disease variance can be attributed to environmental factors such as manual labor, smoking, alcohol use, diabetes, and repetitive hand trauma [5]. It has been suggested that repeated hand-transmitted vibration exposure, particularly in construction and heavy machinery operation, can act as a trigger in genetically predisposed individuals [6,7].

Stenosing tenosynovitis, or trigger finger, is another documented contracture that primarily affects the synovial sheaths of the flexor digitorum tendons [8]. Trigger finger involves inflammation of the synovial sheath of a tendon, causing a disruption in the physiologic pulley system in the fingers [8]. This pulley disruption can result in a flexed finger deformity. Occupational factors that involve gripping, hand-held tools, and repetitive local trauma are frequently reported as contributing to disease onset, though epidemiological links are mixed [9]. The chronic mechanical friction leads to fibrocartilaginous metaplasia, nodule formation and thickening of the effected pulley [9]. Advanced age, high BMI, female sex, and a history of diabetes mellitus or other inflammatory conditions (e.g., rheumatoid arthritis, gout, amyloidosis) were also identified as risk factors for developing trigger finger [10].

Dupuytren’s contracture and stenosing tenosynovitis are well-reported conditions in the literature. However, the incidence of isolated first web space thickening is rarely described. Radial thickening in the setting of Dupuytren’s contracture is not an uncommon finding but is typically associated with concomitant thickening of the ulnar aponeurosis [11]. Other reports describe trauma, burns, and ischemia as factors contributing to first web space contractures [12,13]. Little literature exists describing the case of atraumatic, first web space contracture.

To the authors’ knowledge, we describe the first case of isolated first web space contracture potentially secondary to a repeated stressor. The present report contributes to our current understanding of fibroproliferation of the hand. This report was conducted in accordance with ethical standards laid down in the 1964 Declaration of Helsinki and its later amendments. Informed, written consent was obtained, and ethics committee approval was not required.

## Case presentation

**Figure 1:**
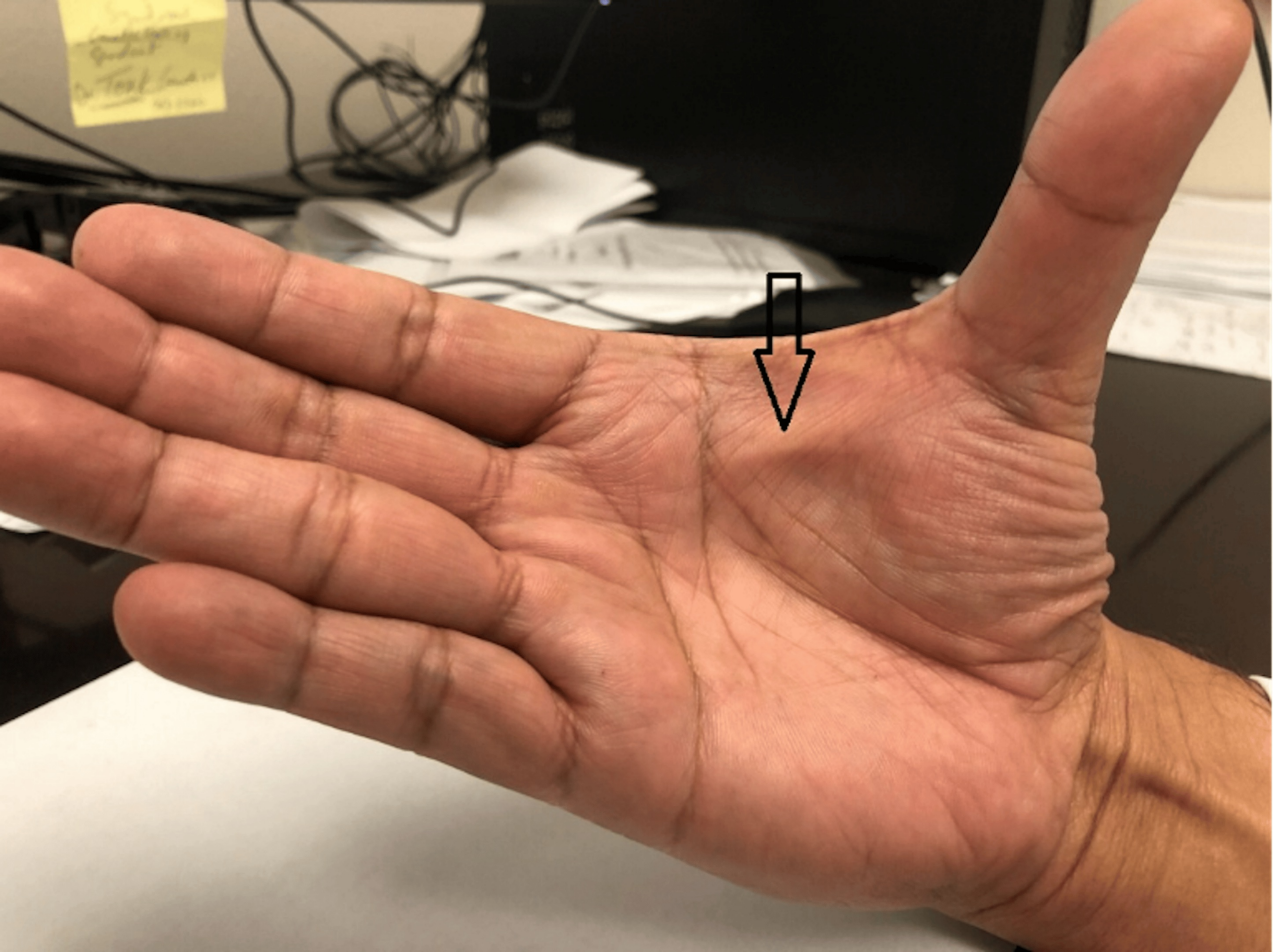
Right-hand image demonstrating gross abnormality of the first web space. The black arrow demonstrates the gross deformity in the first web space of the affected hand.

A 65-year-old right-handed Asian man presented to the clinic with unilateral abnormality of his dominant hand (Figure 1). The patient did not report any pain or functional disability. He is an avid pistol shooter and noted that the abnormality developed since taking up shooting approximately a year prior. There was no history of hand surgery or trauma, alcoholism, diabetes, seizures, palmar/plantar/urogenital contracture, thyroid disease, or family history pertinent to his presentation.

**Figure 2:**
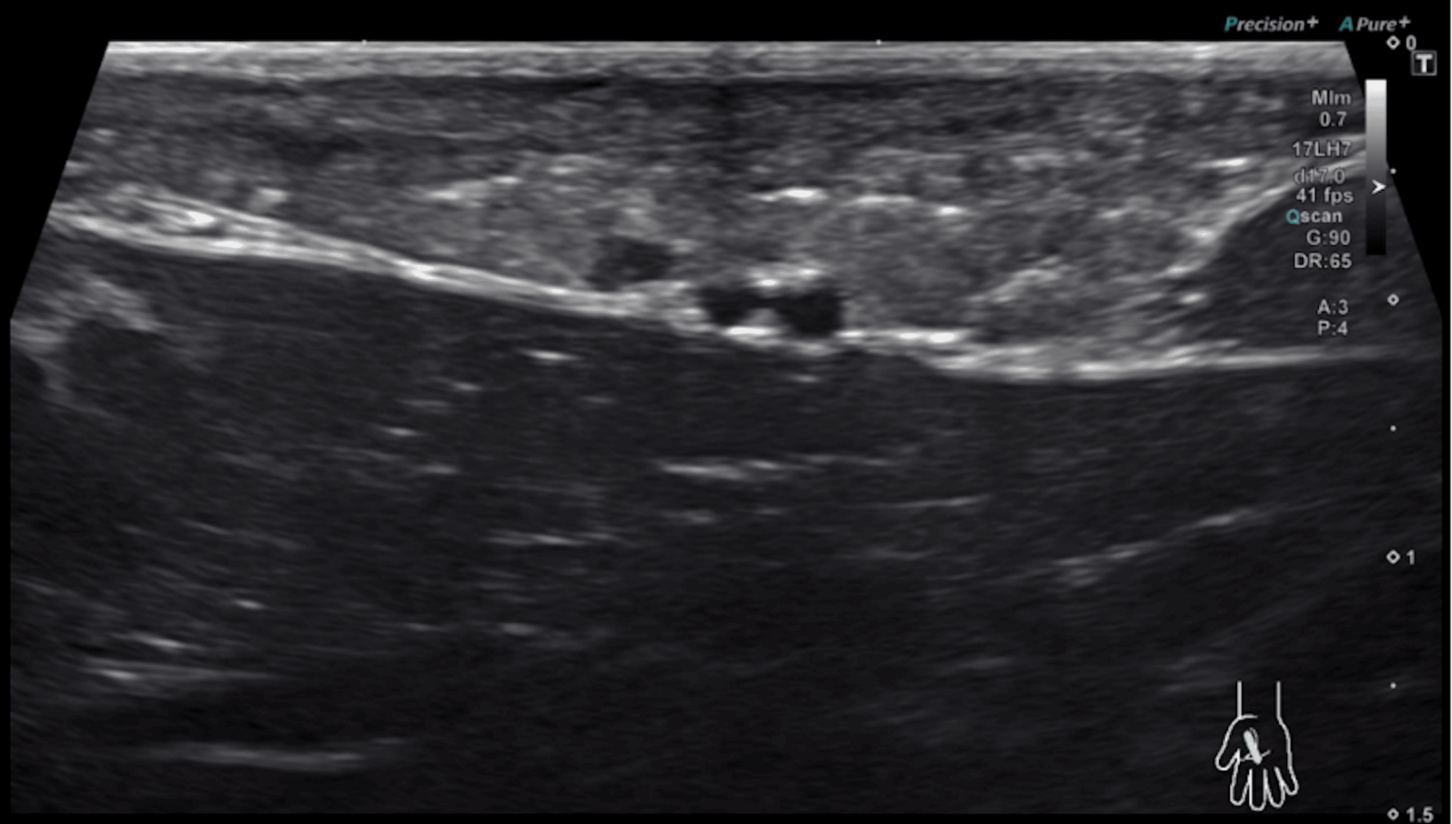
Ultrasound image of the palmar abnormality in longitudinal section This image demonstrates the hyperechoic fibrous thickening of the first web space consistent with fibrotic contracture and loss of normal soft-tissue architecture.

**Figure 3:**
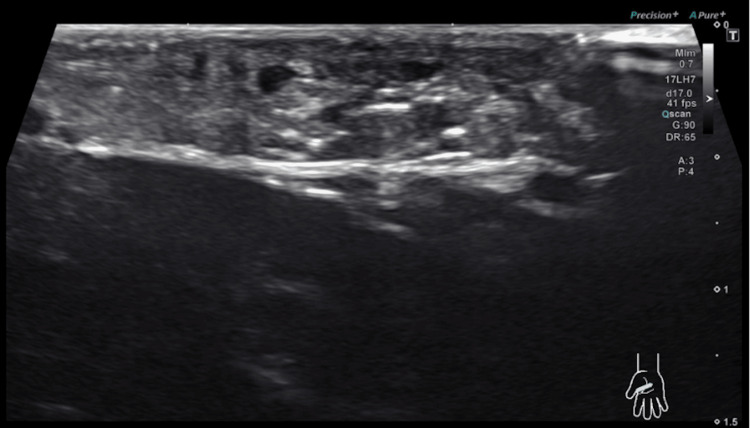
Ultrasound image in the oblique section of the palmar abnormality. This image demonstrates echogenicity and distortion of the normal fascial planes, further supporting a fibrotic contracture of the first web space.

Physical examination revealed a visible, palpable, and thickened cord in the first web space and in line with the lateral margin of the palmar aponeurosis. Examination of the rest of the hand, including the palmar aponeurosis of the second through fourth digits and all synovial sheaths, revealed no further abnormalities. He was neurovascularly intact about the median, ulnar and radial nerve distributions. The cord was not mobile, painful, or inhibitory to hand function. Differential diagnoses at this time included Dupuytren’s contracture and stenosing tenosynovitis. The workup for this patient included an ultrasound, which showed asymmetrical thickening of the radial, lateral edge of the palmar aponeurosis between the first and second digits without evidence of fluid collection or damage to fibrous tissue (Figures 2, 3).

Thus, the findings were felt to represent an atypical pattern of palmar fibroproliferation. Given the history, it is hypothesized that the patient’s palmar thickening was secondary to repetitive recoil exposure from pistol shooting. Given the lack of function-limiting contracture, distal digit involvement, and the isolated first web space lesion, Dupuytren’s contracture and stenosing tenosynovitis are less likely diagnoses.

Given a high clinical suspicion of microtrauma-induced palmar fibroproliferation, histopathologic biopsy was not performed and clinical monitoring was recommended, as the patient was not experiencing pain or functional deficits from the lesion. If the malformation progressed to involve significant contracture or pain, surgical intervention would be considered. To date, the patient has not returned to the clinic for evaluation.

## Discussion

The descriptive working diagnosis of “pistol shooter’s palm” may bear similar name to trigger finger, but it involves thickening of the fascia within the first webspace of the palm. This case represents an important variant from the typical hand contractures of Dupuytren’s or stenosing tenosynovitis and therefore should be addressed. Understanding the similarities and differences of the etiology of pistol shooter’s palm compared to other hand contractures could help improve clinical treatment and prevention [14].

This case is easily distinguishable from stenosing tenosynovitis primarily from the location of the contracture. In trigger finger, inflammation and hypertrophy of the retinacular sheath restrict motion of the flexor tendon, typically at the metacarpophalangeal (MCP) joint [14]. The first flexor tendon at the base of the index finger is the most affected sheath in stenosing tenosynovitis, which is a vaguely similar location to the presented case. However, this patient has contracture of the palmar aponeurosis versus a contracture at the MCP joint.

This case can be distinguished from the classic presentation of Dupuytren’s contracture by multiple features. In “pistol shooter’s palm”, a fibrous band forms between the distal ends of the first and second metacarpals. In comparison, Dupuytren’s contracture is usually observed in the fourth and fifth fingers [3,15]; although first web space involvement is not rare, it is normally associated with concomitant involvement of ulnar-sided pathology [11]. Another noteworthy distinction relevant to this case is the patient’s Asian descent. Dupuytren’s is commonly seen in people of Northern European descent, which underlines the likely genetic predisposition of the disease [3,15,16]. The lack of European ancestry in this case, in combination with the repetitive stress of pistol shooting, suggests a direct environmental etiology as opposed to genetic predisposition. There are studies describing an increased prevalence of Dupuytren’s disease in those with certain occupational exposures, but these studies exclude patients with first web space involvement [6,7].

Regarding fibrotic diathesis, the more aggressive subgroup of Dupuytren’s disease, there are multiple features of this case distinguishing it from this diagnosis as well. Although diathesis typically consists of rapid progression, other factors include bilateral presentation, ectopic lesions and family history of one or more affected siblings or parents [17]. Furthermore, a major distinguishing feature is the earlier age of onset seen with diathesis [17]. The same comorbidities seen in classic Dupuytren’s disease such as diabetes mellitus, excessive alcohol intake, or hepatic disease are prevalent with diathesis as well.

Treatment options for fibroproliferative disease of the palmar aponeurosis include clinical monitoring and surgical intervention. In cases where there is little pain or functional disability, patients and practitioners should monitor for disease progression. As significant contracture causes functional impairment, invasive procedures to release the contracture are warranted. There is still debate regarding the best treatment modality for Dupuytren’s-related contractures, with open fasciectomy providing optimal long-term outcomes and lower recurrence; however, in patients with moderate disease, less invasive options like injectable collagenase or needle fasciotomy can be considered due to easier recovery [18]. To prevent wound-related contracture, open fasciectomy and Z-plasty with a palmar flap has been described [19].

The broader literature suggests that repetitive mechanical stressors may be associated with fibroproliferative changes in the palmar fascia, an association which has been described primarily in occupational and environmental contexts [6,7]. This provides a plausible framework for considering similar mechanisms in recreational activities involving repetitive hand stress. Thus, preventative measures can be recommended in similar cases. Ergonomic strategies aimed at reducing focal pressure and vibration exposure may be reasonable to consider in individuals engaging in such activities. This may include padded or contoured grips, optimization of grip size, recoil-dampening modifications, and limiting of the prolonged repetitive exposure through activity modification. While these strategies are not evidence-based interventions for this specific presentation, they may serve as practical considerations to potentially mitigate localized mechanical stress and support early monitoring rather than unnecessary intervention.

## Conclusions

The present case provides a distinct etiology for fibroproliferation of the palmar aponeurosis, illustrating how repetitive mechanical recoil may contribute to the development of an isolated first web space contracture in the absence of classic risk factors. Given the lack of published cases of isolated first web space contractures, as well as the causative mechanism observed in this case, we believe that this case provides further insight into the development of palmar fibroproliferation. Recognition of this pattern may guide clinicians toward appropriate monitoring rather than unnecessary intervention, and underscores the importance of considering unique occupational or recreational stressors when evaluating palmar fibroproliferative lesions.
